# The Loss of Estradiol by Androgen Deprivation in Prostate Cancer Patients Shows the Importance of Estrogens in Males

**DOI:** 10.1210/jendso/bvae107

**Published:** 2024-06-04

**Authors:** Herjan J T Coelingh Bennink, Amanda Prowse, Jan F M Egberts, Frans M J Debruyne, Ilpo T Huhtaniemi, Bertrand Tombal

**Affiliations:** Pantarhei Bioscience, 3700 AL Zeist, The Netherlands; Terminal 4 Communications, 1217 SK Hilversum, The Netherlands; Terminal 4 Communications, 1217 SK Hilversum, The Netherlands; Andros Clinics, 6842 CV Arnhem, The Netherlands; Institute of Reproductive and Developmental Biology, Imperial College London, London SW7 2AZ, UK; Division of Urology, University Clinic Saint-Luc, 1200 Brussels, Belgium

**Keywords:** androgen deprivation therapy (ADT), bone loss, estetrol (E4), estrogen deficiency symptoms, hot flashes, transdermal estradiol (tE2)

## Abstract

The role of estradiol (E2; an estrogen) in men needs to be more appreciated. In this review, we address the clinical situations that allow the study of the clinical consequences of E2 deficiency in men and discuss the effects of restoration of levels of this reproductive steroid hormone. In men with advanced prostate cancer (PCa) undergoing androgen deprivation therapy (ADT), E2 is suppressed along with testosterone, leading to side effects affecting the quality of life. These include hot flashes, arthralgia, fatigue, mood changes, cognition problems, weight gain, bone loss, and increased risk of cardiovascular disease. Transdermal E2 alone for ADT has shown equivalent testosterone suppression compared to gonadotropin-releasing hormone (GnRH) agonists while also preventing estrogen-deficiency side effects, including hot flashes and bone loss. Co-treatment of ADT with fetal estrogen estetrol (E4) has shown significant improvements of estrogen-deficiency symptoms. These observations emphasize the need to raise awareness of the importance of estrogens in men among clinicians and the lay public.

Testosterone (an androgen) is traditionally considered a “male” hormone and estradiol (E2; an estrogen) a “female” hormone, with essential roles in reproductive and sexual function. Of note, both hormones are produced in both sexes, albeit at very different levels, and the role of estrogens in men (and androgens in women) is becoming increasingly evident. Here, we review the physiology and pathophysiology of estradiol (E2) in males, using the classical basic principle of endocrinology: namely, (i) suppress the hormone; (ii) study the resulting signs and symptoms; (iii) replace the hormone; and (iv) study the effects of restoration. In men with advanced prostate cancer (PCa) treated with androgen deprivation therapy (ADT), E2 is suppressed by more than 80% [1-3], causing severe estrogen deficiency–related signs and symptoms (Table 1) [4, 5]. Estrogens can effectively and safely be restored with transdermal E2 (tE2) or orally with the natural fetal estrogen estetrol (E4) [6, 7].

**Table 1.** Side effects of androgen deprivation therapy with, in brackets, whether it is due to the loss of testosterone (T) or estrogens (E).

“Big 4”	What you see	What is not visible	What the patient feels
Libido loss **(T)**	Weight gain **(E)**	Loss of bone, decreased BMD and increased fracture risk **(E)**	Fatigue (**T & E)**
Erection problems **(T)**	Gynecomastia **(T & E)**	Metabolic syndrome **(E)**	Sleeping problems **(T & E)**
Hot flashes & sweating **(E)**	Muscle atrophy (sarcopenia) **(T & E)**	Anemia **(T)**	Loss of energy **(T & E)**
Arthralgia (joint pain); **(E)**	Decreased erectile function and size of testicles **(T)**	Increased cardiovascular risk **(loss of E)** Inhibition of spermatogenesis and infertility (T)	Apathy **(T & E)**
	Change of hair pattern **(T)**		Mood changes and depression **(E)**
			Cognition and memory problems **(E)**

Adapted from Coelingh Bennink et al 2022 (reproduced according to BY-NC-ND 4.0) [4].

Targeted literature and internet searches were performed (including PubMed). Studies with key evidence regarding the physiology and pathophysiology of E2 in men, and the clinical consequences and therapeutic strategies for the prevention/amelioration of the suppression of E2 in men treated with ADT were identified using a combination of keywords that included *men*, *males*, *estrogen*, *estradiol*, *prostate cancer*, and *androgen deprivation therapy*, together with “pearl growing” citation chasing. Preference is given to studies quantifying E2 or testosterone by mass spectrometry-based methods (LCMS/MS) [8].

##  

### Endocrinological Aspects of E2 in Men

E2 is the most abundant and potent estrogen in men and in women of reproductive age; estrone (E1) is the predominant estrogen in postmenopausal women, estriol (E3) is the main estrogen in pregnant women, while estetrol (E4), which is exclusively produced by the fetal liver, is found in the fetus and at lower levels in the maternal circulation during pregnancy.

In both men and women, E2 is produced from testosterone (T) through aromatization [9]. Ovaries are the primary source of E2 in women; however, in men, 50% to 75% of E2 is produced by extragonadal tissues, including adipose tissue, muscle, bone, and brain, with the remainder being secreted by the testis [10]. E2 levels are considerably lower in men than in women of reproductive age but are similar or even higher than in postmenopausal women (Fig. 1) [11, 12]. E2 levels in men increase during puberty and remain relatively stable through adulthood, although some studies have reported increases with age through middle-age [13-16], with levels decreasing slightly in the elderly [12, 16, 17], in contrast to women where levels decline rapidly and substantially during menopause (Fig. 1). E2 levels have also been reported to increase with increasing body mass index (BMI) in men in some studies [15, 18, 19], while others have reported no association [17, 20]. A large meta-analysis of individual participant data from 9 studies (n = 21 074) found that the association with higher BMI and higher E2 concentrations was only evident in men with a BMI above 32 kg/m^2^ [16]. It has been suggested that increased fat mass in middle and older age in men may explain the stable or increased levels of E2 and a higher E2/T ratio [17, 21], which may be caused by increased aromatase activity in adipose tissue [17]. In addition, E2 levels may differ according to race/ethnicity, with higher E2 levels reported in Black men compared to White men in some studies [18, 22, 23].

**Figure 1.**
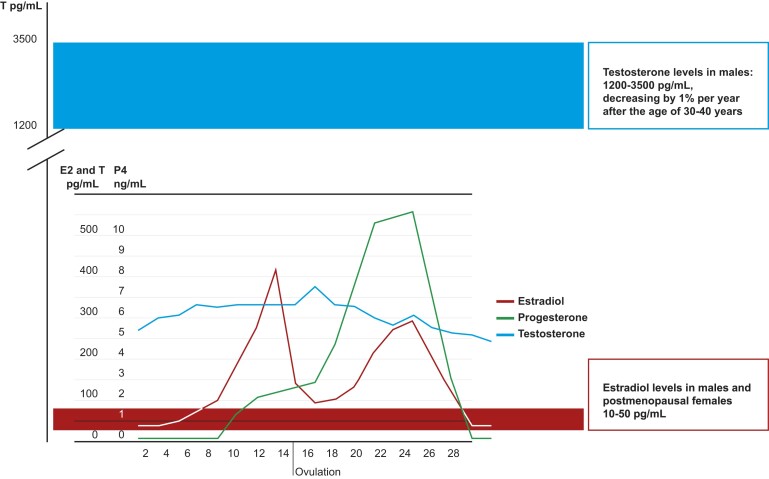
Mean levels of estradiol (E2 pg/mL), progesterone (P4 ng/mL), and testosterone (T pg/mL) during a normal menstrual cycle of 28 days, and corresponding E2 levels in postmenopausal women and in men (red bar) and T levels in men (blue bar). Original figure (sources: Frederiksen et al 2020, and Penell et al 2021) [11, 12].

### Functions of E2 in Men: ADT-Associated Suppression of E2 as a Model

E2 elicits a host of effects across virtually all tissues in the body by binding to specific estrogen receptors (ERs), which in turn regulates an array of transcriptional and epigenetic processes [24-27]. The 3 predominant ERs are ERα, ERβ, and GPER1 (G protein-coupled estrogen receptor 1) [27]. E2 exerts direct “genomic” effects by diffusing into the cell and binding to nuclear ERα and ERβ and this ER complex binds to specific sequences in gene promoters called estrogen response elements (EREs) to activate gene expression. In addition, E2 elicits rapid, nongenomic effects by binding to plasma membrane ERα, ERβ, and GPER1 to induce a diverse array of signaling pathways [27]. In this way, E2 is a key regulator of a multitude of physiological and pathological processes in men as well as in women [10, 28].

The use of ADT to treat advanced PCa is a model to study the role of estrogens in men [3, 4, 10, 29]. Androgens are critical stimulators of PCa, and in men with locally advanced and metastatic PCa, initial treatment is focused on the suppression of androgens by ADT, generally using gonadotropin-releasing hormone (GnRH) agonists (GnRHa) (also known as luteinizing hormone-releasing hormone [LHRH] agonists) or GnRH antagonists as the first-line option [1, 2]. In addition, GnRHa are being increasingly combined with second-generation anti-androgens such as abiraterone, apalutamide, enzalutamide, or darolutamide [1, 2]. Essential for this review is that ADT not only suppresses T levels but also more than 80% of its downstream metabolite E2, causing many and potentially serious signs and symptoms of estrogen deficiency shown in Table 1, and summarized below. The suppression of E2 is an unintended consequence of ADT, and unlike the suppression of T, it is not necessary for the therapeutic benefit of ADT. In fact, prior to the advent of ADT with GnRHa, estrogen was used as a treatment for PCa, inhibiting testicular androgen production and release via a negative feedback loop to inhibit the release of hypothalamic LHRH and pituitary luteinizing hormone (LH) [30].

#### Vasomotor control

Like in women during menopause, vasomotor symptoms (VMS), including hot flashes and sweats, commonly occur in men being treated with ADT. An observational study of 250 PCa patients treated with ADT reported that 80% experienced VMS [31]. VMS-associated sleep disturbance, fatigue, and disruptions to daily activities were commonly reported (25%, 40%, and 40%, respectively), while 12% required further medical intervention to alleviate the symptoms or interrupted treatment [31]. In a clinical trial in healthy men with normal T levels, which used hormonal manipulations to compare the effects of E2 and T on VMS, the incidence of VMS was found to be increased in those with low E2 levels (ie, in those treated with an aromatase inhibitor that suppressed E2 synthesis) even in men with serum T levels above the physiologic range [32], providing support that reduced E2 is responsible for ADT-associated VMS.

#### Bone health

Osteoporosis occurs in up to 53% in men with PCa treated with ADT [33]. A meta-analysis of 5 studies (n = 515) evaluating the effects of ADT on bone mineral density (BMD) in patients with PCa receiving ADT compared to patients with PCa or other urologic conditions not receiving ADT found that ADT was associated with statistically significant decreases of BMD vs the control group in the lumbar spine, femoral neck, and total hip. Bone loss has been shown to be most pronounced in the first year of treatment [34], and it is evident as early as 12 weeks after initiation of ADT [35]. The clinical repercussions are ADT-associated increased risks of fractures and mortality [36, 37]. Insights into the vital role of estrogen signaling in the muscular-skeletal system have come from rare cases of loss of estrogen function due to mutations in the *ERα* gene in men, which result in skeletal and bone defects [38-40]. There is also a wealth of preclinical and clinical evidence showing that E2 is a crucial regulator of various aspects of bone health in men as well as in women [41, 42], as well as recent evidence of a causal effect of E2 on BMD and fracture risk garnered from genetic studies [43-45].

#### Body composition

ADT is also commonly associated with changes in body composition (increases in body weight and percentage fat mass and decreases in percentage lean body mass and muscle size), and an increased risk of sarcopenia and sarcopenic obesity, which in turn is associated with poor outcomes (including impacts on metabolic and cardiovascular diseases, described below) and adverse impacts on health-related quality of life (HRQoL) [46-49]. Regarding E2 deficiency in men and changes in fat mass, a rare case of mutations that resulted in a lack of aromatase activity and undetectable E2 in a man was characterized clinically by abdominal obesity, hyperinsulinemia, acanthosis nigricans, and nonalcoholic fatty liver disease [50]. The mechanisms of the protective effects of E2 against obesity are still being elucidated, but they may involve regulation of glucose-insulin homeostasis and leptin, affecting food intake and energy expenditure. ERα-knockout male and female mice show large increases in white adipose tissue together with insulin resistance and glucose intolerance in both sexes, associated with lower energy expenditure [51, 52]; male and female aromatase-knockout mice show a similar phenotype with increased adiposity associated with reduced spontaneous physical activity levels, reduced glucose oxidation, and a decrease in lean body mass [53]. Treatment with E2 has been shown to protect against obesity and alterations in glucose-insulin homeostasis in male mice fed high-fat diets [54], and to regulate leptin sensitivity to modulate feeding [55]. Of note, clinical association studies (ie, measuring E2 levels with increasing fat mass/BMI) are complex because with increasing fat mass and resulting increases in aromatization, E2 levels can in turn increase [17], with a positive association between E2 levels and increasing adiposity being reported in some studies [15, 18, 19]. Of note, there have also been inconsistent results in studies evaluating the effect of hormone replacement therapy (HRT) on glucose homeostasis, weight, adiposity, and diabetes in menopausal women, further highlighting the complex relationship between E2 and body composition [56]. However, a consensus is emerging that estrogen replacement may be beneficial for β-cell insulin secretion, glucose effectiveness, and insulin sensitivity, decreasing the risk of type 2 diabetes [56].

#### Metabolic and cardiovascular diseases

Type 2 diabetes and cardiovascular disease (CVD) are also common comorbidities in patients with PCa. In addition, there is evidence that ADT may increase the risk of these diseases. Since 2010 the Food and Drug Administration (FDA) has required the label for GnRHa to carry a warning for an increased risk of diabetes and certain CVDs (heart attack, sudden cardiac death, and stroke). Systematic reviews have found a relatively consistent association between ADT and an increased risk of diabetes [57]. In contrast, the data of an ADT-associated increased risk of CVD have been less consistent [58-60]. The complex mechanisms underlying these (potential) associations are still being elucidated. One possible explanation is that GnRHa suppress LH but are less effective in inhibiting follicle-stimulating hormone (FSH), a hormone that has been implicated in the development or worsening of atherosclerotic plaques, increased adiposity, metabolic syndrome, and insulin resistance [61, 62].

Regarding E2, there is a wealth of molecular, clinical, and epidemiological evidence of it eliciting protective cardiovascular and metabolic properties in women. Premenopausal women have a reduced risk of type 2 diabetes and CVD compared with age-matched men, but this advantage is lost after menopause. Mechanistically, E2 appears to have numerous effects in a range of tissues which influence energy balance, weight homeostasis and obesity (described above), glucose homeostasis, lipid profiles, the gut microbiome, vascular and gut inflammation, and vascular endothelial function [63-66]; however, it is not yet clear which of these effects (if any) are primarily related to the GnRHa-associated increased risk of diabetes and (potentially) CVD. Interestingly, recent data have implicated sex hormone ratios (the ratio of estrogen to testosterone as opposed the levels of individual hormones in isolation) as being important for the modulation of metabolic parameters and CVD in men [67-70].

#### Joint pain

Joint pain is a frequently occurring and heavily underestimated consequence of ADT treatment. A recent study demonstrated that PCa patients receiving ADT showed significantly higher incidence rates of hand joint symptoms and hand joint pain compared to hormone-naïve PCa patients [71].

#### Rheumatoid arthritis

Two large cohort studies (n = 100 000 and n = 12 505 patients with PCa) have reported that ADT use is associated with an increased risk of rheumatoid arthritis (RA) [72, 73]. The mechanisms of ADT-associated increases in joint pain and RA in men with PCa have yet to be elucidated, although sex hormones, including testosterone due to its immunosuppressive effects, FSH, and estrogens have been implicated [74-76]. “Female” hormones have been implicated in RA in women; in the general population RA is much more common in women, generally starting or worsening during (peri)menopause when E2 levels decrease [77]. Furthermore, early age at menopause, the postpartum period and the use of anti-estrogen agents are associated with RA onset or flare, while pregnancy is protective [77]. In addition, experimental studies have suggested that E2 elicits protective immunomodulatory, anti-inflammatory, autophagic and apoptotic properties in in vitro and in vivo models of RA [78-82].

#### Cognitive dysfunction

According to a recent review, studies have reported that between 10% and 70% of patients with PCa treated with ADT experience cognitive impairment [83]. A number of large cohort studies (using data from registries or healthcare databases) have evaluated whether ADT is associated with cognitive dysfunction but have reported conflicting results [84-86]. Recent systematic reviews and meta-analyses have suggested that second-generation anti-androgens may be associated with negative cognitive effects [87, 88]. Mechanistically, there is evidence that E2 has neuroprotective properties, mediated via the cholinergic system, the dopaminergic system, the mitochondrial bioenergetic system and/or the immune system [89]. In women, E2 has been implicated in cognitive dysfunction: cognitive symptoms are commonly reported during (peri-)menopause [90] and early menopause and premature ovarian insufficiency have been associated with an increased risk of dementia [91]. In addition, E2 levels after menopause have been inversely associated with Alzheimer disease and cognitive impairment [92]. In the 18-year follow-up of the Women's Health Initiative (WHI) study focusing on mortality, death due to Alzheimer disease was decreased by 26% in the estrogen-only treatment group compared to placebo [93]. Furthermore, dementia is more common in older women than older men [94], which may partly be explained by lifespan, but there is also evidence of sex-specific differences in neuropathological processes that may be mediated in part by sex hormones, including E2 [95-97]. However, to date, E2's role in cognition in men with PCa treated with ADT has been less well-studied.

### Restoration of Estrogen in Advanced Prostate Cancer Patients Treated With Androgen Deprivation Therapy

Before the advent of GnRH analogs, potent synthetic estrogens such as diethylstilbestrol (DES) were successfully used for the treatment of PCa, but they were associated with cardiovascular thromboembolic events [30, 98], and therefore estrogens were abandoned. However, safer estrogen formulations, especially transdermal E2 (tE2) and the natural fetal estrogen estetrol (E4) are now being evaluated as a method for alleviating the estrogen-related side effects of ADT, with the potential to also exert anticancer effects by further inhibition of total and free T, prostate-specific antigen (PSA), FSH, and insulin-like growth factor 1 (IGF-1) [6, 99]. Regarding FSH, it is worth noting that there are no definitive data suggesting that FSH stimulates IGF-1, and neurokinin 3 receptor antagonists, known to improve hot flash symptoms in menopausal women, do not affect FSH and E2 levels [100]. It may even be possible, provided that the gonadotropin and testosterone suppression is sufficient, to replace GnRH analogue ADT with tE2 or E4, thereby integrating the estrogen substitution issue with the suppression of testicular androgen production.

### Transdermal E2

There is growing evidence that the use of tE2 alone as ADT can reach castrate T levels [101] and may not be associated with the cardiovascular thromboembolic events associated with oral administration of high doses of E2, by avoiding the oral first-pass hepatic metabolism involved in the activation of coagulation pathways by E2 [102, 103].

In the ongoing phase 2/3, randomized PATCH study, tE2 alone is being evaluated in comparison to treatment with GnRHa, not as an add-on, with the premise that E2 itself can work as the androgen-suppressing therapy without the estrogen-deficiency side effects associated with GnRHa or the cardiovascular thromboembolic side effects of oral E2 [101, 104, 105]. Long-term data (median follow-up of 3.9 years) from 790 patients receiving GnRHa and 904 receiving tE2 (administered as a patch) showed no difference in either overall or fatal cardiovascular events (Table 2) [101]. At 6 months of treatment, patients treated with tE2 reported better HRQoL (EORTC-QLQ-C30) associated with less fatigue, better physical function, fewer hot flashes, and improved sexual activity and interest in sex, although gynecomastia was more common with tE2 (Table 2) [104]. Furthermore, tE2 was associated with improvements in BMD (measured at 1 and 2 years of treatment) compared to treatment with a GnRHa (Table 2) [105]. Crucial to the approach of using tE2 alone (ie, not as add-on to ADT) is to show that tE2 has a noninferior efficacy to GnRHa. While castration rates (ie, T levels ≤ 1.7 nmol/L) at 1 and 3 months have been shown to be similar between groups [101] (Table 2), we need to await overall survival and progression-free survival data of tE2 compared to GnRHa, anticipated in 2023/2024, to determine whether tE2 alone is as effective as GnRHa as a viable treatment option. To complement the PATCH trial, further evidence may also come from the STAMPEDE trial (NCT00268476), a large multi-arm adaptive trial, has been recruiting patients into an tE2 arm since 2017, with the aim of performing a meta-analysis of the results across the 2 trials [106].

**Table 2.** Results of the PCombi and PATCH studies evaluating estrogens for the treatment of prostate cancer and ADT-induced estrogen deficiency–related side effects

Outcomes	PATCH: tE2 (n = 940) vs GnRHa (n = 790) [101, 104, 105] Long-term seamless phase 2/3, randomized, active-controlled, multicenter trial NCT00303784	Russell et al: tE2 + GnRHa*^a^* (n = 39) vs placebo + GnRHa*^a^* (n = 39) [7, 108] 6-month, double-blind, randomized, placebo-controlled trial ACTRN12614000689673	PCombi [6, 114]: E4 + LHRHa (n = 37) vs placebo + LHRHa (n = 20) [6, 114] 24-week phase 2, double-blind, multicenter, randomized, placebo-controlled trial NCT03361969
PCa parameters	Castration rate at 1 month: 83% vs 65% Castration rate at 3 months: 93% vs 93%	NR	Castration rate from week 4 through week 24): 100% vs 100% PSA levels: % of patients with PSA < 0.5 ng/mL at week 24: 84% vs 45% LH: mean (SD) change from baseline to week 24: −97.6 (1.8) vs −97.6 (2.8) IU/L FSH: mean (SD) change from baseline to week 24: −97.8 (1.7) vs −56.7 (44.8) IU/L
Hot flashes (% patients)	6 months: “Quite a bit” or “very much” hot flashes: 8% vs 46%, *P* < .001	Median (IQR) at 6 months, mean adjusted difference (95% CI) Daily hot flash frequency: 0.7 (0.0; 2.7) vs 2.7 (0.0; 6.8), −1.6 (−2.7 to −0.5), *P* = .04 Weekly hot flash score: 5.0 (0.0; 19.0) vs 21.5 (0.5; 69.2), −19.6 (−35.5 to −3.8), *P* = .11	At least one hot flash during week 23: 14.3% vs 60.0%, *P* < .001 Daily hot flashes at week 24: 5.9% vs 55%
Bone parameters	Mean (SD) change from baseline in BMD at 1 year Lumbar spine: + 0.069 (0.076) vs −0.021 (0.057), *P* < .001 Right hip: + 0.016 (0.049) vs −0.022 (0.033), *P* = .003 Left hip: + 0.019 (0.055) vs −0.026 (0.016), *P* = .002 Whole body: + 0.017 (0.026) vs −0.015 (0.043), *P* = .002 Mean (SD) change from baseline in BMD at 2 year Lumbar spine: + 0.077 (0.060) vs −0.026 (0.086), *P* = .001 Right hip: + 0.017 (0.044) vs −0.040 (0.070), *P* = .002 Left hip: + 0.018 (0.031) vs −0.047 (0.036), *P* < .001 Whole body: + 0.002 (0.069) vs −0.089 (0.106), *P* = .006	Mean adjusted difference vs placebo at 6 months Total vBMD at the distal tibia: 2.0 mgHA/cm^3^ (95% CI: −0.8 to 4.8), *P* = .17 Cortical vBMD at the distal radius: 14.8 mgHA/cm^3^ (95% CI: 4.5 to 25.0), *P* = .005 Failure load at tibia: 250 N (95% CI: 36 to 465), *P* = .02 Failure load at radius: 193 N (95% CI: 65 to 320), *P* = .003 aBMD at the lumbar spine: 0.02 g/cm^2^ (95% CI: 0.01 to 0.03), *P* = .01 aBMD at ultra-distal radius: 0.01 g/cm^2^ (95% CI: 0.00 to 0.02), *P* = .01 Bone remodeling markers CTX: *P* < .0001 vs placebo P1NP: *P* < .005 vs placebo	Osteocalcin: Mean (SD) change from baseline to week 24: −22.0 (19.7) vs +47.6 (47.2) ng/mL, *P* < .0001 CTX1: Mean (SD) change from baseline to week 24: −24.8 (34.6) vs +151.1 (109.1) ng/mL, *P* < .0001
Other key complaints (% patients)	Lack of sexual interest: 59% vs 74% “Not at all” sexually active: 78% vs 87%	NR	Night sweats: 2.9% vs 30.0% Fatigue: 29.4% vs 50.0% Arthralgia: 17.7% vs 40.0%
HRQoL	EORTC-QLQ-C30 mean change from baseline to 6 months (95% CI): Global score: −2.8 (−4.7, −0.8) vs −5.0 (−7.4, −2.7), *P* = .006 Physical function: −2.8 (−4.7, −1.0) vs −5.7 (−7.9, −3.5), *P* < .001 Fatigue: 6.0 (3.7, 8.2) vs 8.3 (5.8, 10.8), *P* = .02 Cognitive function: −3.5 (−5.3, −1.6) vs −4.1 (−6.2, −2.0), *P* = .32	Mean (IQR) at 6 months FACT-P Total: 129 (114; 136) vs 124 (110;134) FACT-G Total: 94 (86; 98) vs 93 (81; 97) AMS Total: 38 (32; 42) vs 40 (32; 48)	Mean (SD) at week 24 FACT-P Total: 122.2 (12.3) vs 118.7 (19.7) FACT-P Trial Outcome Index: 81.4 (9.4) vs 79.1 (13.6) FACT-G Total: 85.5 (11.7) vs 86.6 (11.4) FACT-G Prostate Cancer Subscale: 34.5 (4.77) vs 34.2 (9.96) FACT-G Physical Well-Being: 25.7 (2.6) vs 25.2 (3.6) FACT-G Functional Well-Being: 21.0 (3.7) vs 19.7 (4.8)
CV events (% patients)	Median follow-up 3.9 (IQR 2.4-7.0) years: 10% vs 8%	VTE: 0 vs 0 Acute coronary syndrome: 0 vs 0 Stroke: 0 vs 0	12.2% vs 9.5%
Most common AEs (% patients)	Median follow-up 3.9 (IQR 2.4-7.0) years: Gynecomastia: 86% vs 38% Hot flashes: 35% vs 86% Skin or subcutaneous toxicity: 68% vs 65% Sexual or reproductive toxicity: 91% vs 92%	Gynecomastia: 44% vs 21% Nipple tenderness: 28% vs 3% Rash: 10% vs 21%	Hot flashes: 29.3% vs 52.4% Nipple pain: 34.1% vs 0% Gynecomastia: 17.1% vs 0%

EORTC-QLQ-C30: For global quality of life, cognitive function, and physical function, a higher score corresponds to a better outcome. For fatigue, a higher score corresponds to more fatigue. Castration rate = % of patients with suppression of total T below castrate levels of 1.7 nmol/L (50 ng/dL).

Abbreviations: ADT, androgen deprivation therapy (luteinizing hormone-releasing hormone agonist or gonadotropin-releasing hormone antagonists); AE, adverse event; AMS, Aging Males’ Symptoms scale (possible range 17-85; lower scores better); BMD (a or v), bone mineral density (volumetric or areal); CV, cardiovascular; FACT-G, Functional Assessment of Cancer Therapy-General (possible range 0-108; higher scores better); FACT-P, Functional Assessment of Cancer Therapy -Prostate (possible range 0-156; higher scores better); FSH, follicle-stimulating hormone; GnRHa, gonadotropin-releasing hormone agonist; HRQoL, health-related quality of life; IQR, interquartile range; LH, luteinizing hormone; LHRHa, luteinizing hormone-releasing hormone agonist; PSA, prostate-specific antigen; VTE, venous thromboembolism.

^*a*^In the study by Russell et al, ADT was with either a gonadotropin-releasing hormone agonist or antagonist.

The results from the PATCH study are in-line with another smaller study that has evaluated tE2 gel as an add-on to ADT with GnRH agonists or GnRH antagonists (n = 39) compared to placebo + ADT (n = 39) (Table 2). This study is different to the PATCH study in that the tE2 is given in addition to ADT. This trial has also shown tE2-associated improvements in the number and severity of hot flashes and measures of BMD and bone strength compared to ADT with GnRH agonists or antagonists (Table 2) [7, 107, 108]. The tE2 showed no significant effects, neither positive or negative, on HRQoL measured using the Aging Males’ Symptoms scale, the Functional Assessment of Cancer Therapy-General (FACT-G) and FACT-Prostate (FACT-P) [108]. tE2 also showed no significant effects on cognition including verbal learning and memory (International Shopping List test) and spatial problem solving (Groton Maze Learning test), and anxiety and depression (Hospital Anxiety and Depression Scale [HADS]), although the study was not powered to detect differences and the participants had not been selected for having cognitive dysfunction at baseline [109]; therefore, further studies assessing this outcome are warranted. Also of interest is that tE2 resulted in nonsignificant trends for increases in total and regional fat mass, which was contrary to expectations based on data showing that E2 may be involved in preventing obesity (described above) [7]. The authors hypothesized that T may be required for E2-mediated effects on fat [7].

### Estetrol

Estetrol (E4) has several favorable properties, including high oral bioavailability, a lack of active or toxic metabolites, a long oral elimination half-life, a favorable safety profile, especially a better cardiovascular safety profile, a low impact on coagulation and other hemostatic liver factors, and possible beneficial effects on lipids, carbohydrate metabolism, and bone turnover [110-113]. Estetrol is the estrogen in a new E4/drospirenone oral contraceptive, and it is also being evaluated for hormone replacement therapy (HRT), advanced breast cancer treatment, and as described here, for advanced PCa [112].

In the phase 2, double-blind, randomized, placebo-controlled PCombi study, 57 patients with locally advanced or metastatic PCa received high-dose E4 (HDE4; N = 37) or placebo (N = 20) in addition to ADT with a LHRH agonist [6, 114]. The PCombi study is also different from the PATCH study in that the estrogen is given in addition to ADT. Compared to placebo + LHRHa, HDE4 + LHRHa was associated with significant reductions in the number and severity of hot flashes and in bone turnover parameters (Table 2) [6]. HDE4 treatment was also associated with a lower incidence of night sweats, arthralgia, and fatigue, and patients reported an improvement in HRQoL (improvements in the FACT-P total score as well as various FACT subscales) [114]. Furthermore, HDE4 enhanced the suppression of total T, free T, PSA, FSH (LH was already completely suppressed by ADT), and IGF-1, suggesting that E4 has the potential to elicit dual efficacy by (i) reducing the estrogenic side effects of ADT; while (ii) further increasing the antitumor activity of ADT (Fig. 2) [6]. Nipple tenderness and gynecomastia were more common in the HDE4 group, but these adverse events did not result in treatment discontinuations [6].

**Figure 2.**
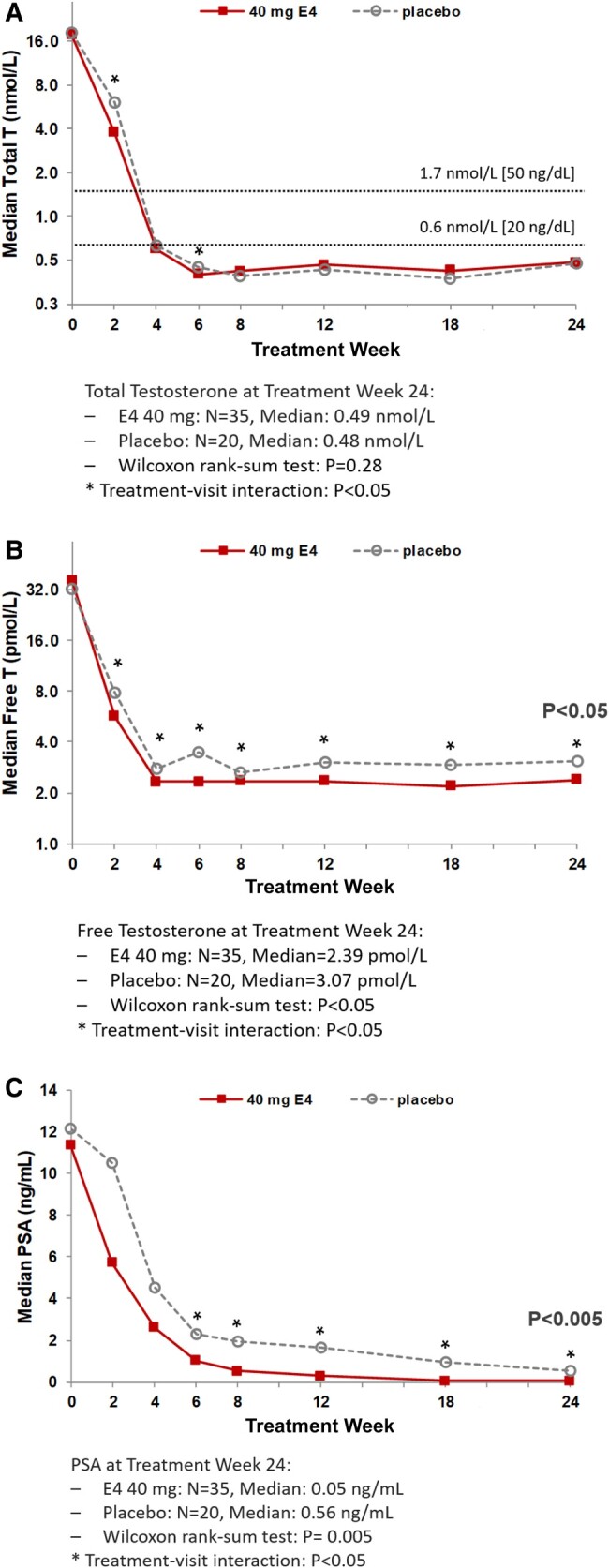
Median levels of (A) total testosterone, (B) free testosterone, and (C) prostate-specific antigen (PSA) after 2, 4, 6, 8, 12, 18, and 24 weeks of treatment with 40 mg estetrol (E4) or placebo in patients with prostate cancer treated with a GnRH agonist [6]. Note: E2, estradiol; P4, progesterone; T, testosterone. Adapted from Coelingh Bennink et al 2021 (reproduced according to BY-NC-ND 4.0) [6].

## Conclusions

The use of ADT for advanced PCa is an endocrine model to study the role of estrogens in men since the suppression of testosterone causes the loss of estradiol. For decades, men with advanced PCa have been exposed to the ADT-induced severe estrogen deficiency–related side effects in exchange for increased survival. Restoring estrogen function by using the natural estrogens transdermal estradiol or the oral fetal estrogen estetrol may alleviate estrogen-related side effects, such as hot flashes, arthralgia, fatigue, mood changes, cognition problems, and bone loss, while also treating PCa more effectively without the adverse cardiovascular effects of previous older estrogens.

**Highlights**

- ADT for prostate cancer treatment suppresses E2 and causes serious estrogen-deficiency symptoms.
- ADT-associated estrogen-deficiency symptoms include hot flashes, arthralgia, and bone loss.
- ADT by transdermal E2 or ADT combined with E4 prevents/treats estrogen-deficiency symptoms.

## Data Availability

All data generated or analyzed during this study are included in this published article.

## Abbreviations

ADT: androgen deprivation therapy
BMD: bone mineral density
BMI: body mass index
CVD: cardiovascular disease
E1: estrone
E2: estradiol
E4: estetrol
ER: estrogen receptor
ERE: estrogen response element
GnRH: gonadotropin-releasing hormone
GnRHa: gonadotropin-releasing hormone agonists
GPER: G-protein coupled estrogen receptor
HDE4: high-dose estetrol
HRQoL: health-related quality of life
IGF-1: insulin-like growth factor 1
LH: luteinizing hormone
LHRH: luteinizing hormone-releasing hormone
PCa: prostate cancer
PSA: prostate-specific antigen
RA: rheumatoid arthritis
T: testosterone
tE2: transdermal estradiol
VMS: vasomotor symptoms
